# Keratinocyte expression of inflammatory mediators plays a crucial role in substance P-induced acute and chronic pain

**DOI:** 10.1186/1742-2094-9-181

**Published:** 2012-07-23

**Authors:** Tzuping Wei, Tian-Zhi Guo, Wen-Wu Li, Saiyun Hou, Wade S Kingery, John David Clark

**Affiliations:** 1 Physical Medicine and Rehabilitation Service, Veterans Affairs Palo Alto Health Care System, Palo Alto, CA94304, USA; 2 Anesthesiology Service, Veterans Affairs Palo Alto Health Care System, 3801 Miranda Avenue (112-A), Palo Alto, CA94304, USA; 3 Department of Anesthesiolgy, Stanford University School of Medicine, Stanford, CA94304, USA

## Abstract

Tibia fracture in rats followed by cast immobilization leads to nociceptive, trophic, vascular and bone-related changes similar to those seen in Complex Regional Pain Syndrome (CRPS). Substance P (SP) mediated neurogenic inflammation may be responsible for some of the signs of CRPS in humans. We therefore hypothesized that SP acting through the SP receptor (NK1) leads to the CRPS-like changes found in the rat model. In the present study, we intradermally injected rats with SP and monitored hindpaw mechanical allodynia, temperature, and thickness as well as tissue levels of tumor necrosis factor-α (TNF-α), interleukin 1β (IL-1β), interleukin 6 (IL-6), and nerve growth factor-β (NGF) for 72 h. Anti-NGF antibody was utilized to block the effects of SP-induced NGF up-regulation. Fracture rats treated with the selective NK1 receptor antagonist LY303870 prior to cast removal were assessed for BrdU, a DNA synthesis marker, incorporation in skin cells to examine cellular proliferation. Bone microarchitecture was measured using micro computed tomography (μCT). We observed that: (1) SP intraplantar injection induced mechanical allodynia, warmth and edema as well as the expression of nociceptive mediators in the hindpaw skin of normal rats, (2) LY303870 administered intraperitoneally after fracture attenuated allodynia, hindpaw unweighting, warmth, and edema, as well as cytokine and NGF expression, (3) LY303870 blocked fracture-induced epidermal thickening and BrdU incorporation after fracture, (4) anti-NGF antibody blocked SP-induced allodynia but not warmth or edema, and (5) LY303870 had no effect on bone microarchitecture. Collectively our data indicate that SP acting through NK1 receptors supports the nociceptive and vascular components of CRPS, but not the bone-related changes.

## Introduction

Complex regional pain syndrome (CRPS) is a painful, disabling and often chronic condition affecting the extremities and is a frequent sequela of tibial and radial fractures [1]. Previously we described a distal tibial fracture model in rats that exhibits chronic unilateral hindlimb warmth, edema, facilitated spontaneous protein extravasation, allodynia, postural unweighting, and periarticular osteoporosis [2]. These post-fracture changes closely resemble the clinical presentation of patients with acute CRPS.

The inflamed appearance of the limb affected by CRPS has led to the hypothesis that the local production of inflammatory mediators might be involved in the etiology of the condition. There is increased TNF-α and IL-6 in blister fluid from patients with early CRPS [3]. Similarly, we have observed a dramatic increase in hindpaw skin expression of TNF-α, IL-1β, IL-6, and nerve growth factor (NGF) at both the mRNA and protein levels [4-6] in the rat fracture model. Treating fractured rats with a TNF-α inhibitor (etanercept), an IL-1 receptor antagonist (anakinra), or an anti-NGF antibody (tanezumab) reduced hindpaw allodynia and unweighting at 4 weeks post-fracture [4,5,7]. These data indicate that fracture-induced allodynia can be attributed partially to local inflammatory mediators because all these drugs are large molecular weight proteins that cannot cross the blood brain barrier. Recently we identified keratinocytes in the fracture-affected dorsal hindpaw as the primary cellular source of the inflammatory nociceptive mediators TNF-α, IL-1β, IL-6, and NGF in the rat fracture CRPS model [8].

Several lines of clinical investigation support the hypothesis that facilitated peripheral neurogenic inflammation, involving neuropeptides such as substance P (SP), contributes to some of the signs and symptoms of CRPS [9-12]. When SP is microdialyzed in the skin of normal volunteers and patients with CRPS, much greater protein extravasation is observed in CRPS-affected limbs, indicating post-junctional facilitation of the SP extravasation response [11,13]. Furthermore, tibial fracture in rats upregulates NK1 receptor expression in skin (keratinocytes) and microvasculature (endothelial cells) of the affected hindpaw [14], and SP signaling is enhanced in the injured limb of these animals [2,14,15]. Treatment with the selective NK1 antagonist LY303870 attenuated spontaneous protein extravasation, edema, warmth, and allodynia in the hindpaw after fracture [2]. SP can induce keratinocyte proliferation and activation *in vitro*[16,17], and in rats electrical stimulation of the sciatic nerve at intensities causing neuropeptide release leads to a gradual increase in hindpaw skin levels of TNF-α, IL-1β, IL-6, and NGF [18]. When SP or calcitonin gene-related peptide (CGRP) was microdialyzed in the skin of normal volunteers there was no immediate pain response [13]. These data support the premise that SP and CGRP act as intermediate mediators in the development of inflammatory pain via other downstream substances such as cytokines and NGF.

In light of these observations, we hypothesized that: 1) exogenous SP signaling in the skin can sequentially upregulate the expression of TNF-α, IL-1β, IL-6, and NGF, resulting in hindpaw nociceptor sensitization, allodynia and unweighting; 2) NGF is the final downstream inflammatory mediator for SP-induced cutaneous nociceptive sensitization; 3) endogenous SP signaling in the fractured limb activates keratinocytes and upregulates expression of TNF-α, IL-1β, IL-6, and NGF, resulting in hindpaw nociceptor sensitization, allodynia and unweighting, and 4) SP signaling after fracture induces periarticular bone loss. The experiments described in this study were designed to test these hypotheses.

## Materials and methods

These experiments were approved by our institute’s Subcommittee on Animal Studies and followed the guidelines of the International Association for the Study of Pain (IASP) [19]. Adult (9-month-old) male Sprague Dawley rats (Simonsen Laboratories, Gilroy, CA, USA) were used in all experiments. The animals were housed individually in isolator cages with solid floors covered with 3 cm of soft bedding and were fed and watered *ad libitum*. During the experimental period the animals were fed Lab Diet 5012 (PMI Nutrition Institute, Richmond, IN, USA), which contained 1.0% calcium, 0.5% phosphorus, and 3.3 IU/g of vitamin D3, and were kept under standard conditions with a 12-h light–dark cycle.

### Surgery

Tibial fracture was performed under isoflurane anesthesia as we have previously described [2]. The right hindlimb was wrapped in stockinet (2.5 cm wide) and the distal tibia was fractured using pliers with an adjustable stop that had been modified with a 3-point jaw. The hindlimb was wrapped in casting tape so the hip, knee and ankle were flexed. The cast extended from the metatarsals of the hindpaw up to a spica formed around the abdomen. To prevent the animals from chewing at their casts, the cast material was wrapped in galvanized wire mesh. The rats were given subcutaneous saline and buprenorphine (0.03 mg/kg) immediately after the procedure and on the next day after fracture for postoperative hydration and analgesia. At 4 weeks the rats were anesthetized with isoflurane and the cast removed with a vibrating cast saw. All rats used in this study had union at the fracture site after 4 weeks of casting.

### Drugs

The anti-NGF antibody muMab 911 (Rinat Laboratories, Pfizer Inc, SF, CA, USA) is a TrkA immunoglobulin G (TrkA-IGG) fusion molecule that binds to the NGF molecule, thus blocking the binding of NGF to the TrkA and p75 NGF receptors and inhibiting TrkA autophosphorylation [20]. Pharmacokinetic and behavioral experiments in rodents indicate that muMab 911 has a terminal half-life of 5 to 6 days in plasma and that a 10 mg/kg dose administered every 5 or 6 days reduces nociceptive behavior in a variety of rodent chronic pain models [21-23].

The NK1 receptor antagonist LY303870 was a generous gift from Dr. L. Phebus (Eli Lily Company, Indianapolis, IN, USA). This compound has nanomolar affinity for the rat NK1 receptor, has no affinity for 65 other receptors and ion channels, has no sedative, cardiovascular or core body temperature effects in rats at systemic doses up to 30 mg/kg, and is physiologically active for 24 h after a single systemic dose of 10 mg/kg [24-26].

### Hindpaw nociception

To measure mechanical allodynia in the rats an up-down von Frey testing paradigm was used as we have previously described [2,15,27]. Rats were placed in a clear plastic cylinder (20 cm in diameter) with a wire mesh bottom and allowed to acclimate for 15 minutes. The paw was tested with one of a series of eight von Frey hairs ranging in stiffness from 0.41 g to 15.14 g. The von Frey hair was applied against the hindpaw plantar skin at approximately midsole, taking care to avoid the tori pads. The fiber was pushed until it slightly bowed and then it was jiggled in that position for 6 seconds. Stimuli were presented at an interval of several seconds. Hindpaw withdrawal from the fiber was considered a positive response. The initial fiber presentation was 2.1 g and the fibers were presented according to the up-down method of Dixon to generate six responses in the immediate vicinity of the 50% threshold. Stimuli were presented at an interval of several seconds. An incapacitance device (IITC Inc. Life Science, Woodland, CA, USA) was used to measure hindpaw unweighting. The rats were manually held in a vertical position over the apparatus with the hindpaws resting on separate metal scale plates and the entire weight of the rat was supported on the hindpaws. The duration of each measurement was 6 s and 10 consecutive measurements were taken at 60-second intervals. Eight readings (excluding the highest and lowest ones) were averaged to calculate the bilateral hindpaw weight-bearing values.

### Hindpaw temperature

The room temperature was maintained at 23°C and humidity ranged between 25 and 45%. The temperature of the hindpaw was measured using a fine wire thermocouple (Omega, Stanford, CT, USA) applied to the paw skin, as previously described [2,15,27]. The investigator held the thermistor wire using an insulating Styrofoam block. Three sites were tested over the dorsum of the hindpaw; the space between the first and second metatarsals (medial), the second and third metatarsals (central), and the fourth and fifth metatarsals (lateral). After a site was tested in one hindpaw the same site was immediately tested in the contralateral hindpaw. The testing protocol was medial dorsum right then left, central dorsum right then left, lateral dorsum right then left, medial dorsum left then right, central dorsum left then right, and lateral dorsum left then right. The six measurements for each hindpaw were averaged for the mean temperature.

### Hindpaw thickness

A laser sensor technique was used to determine the dorsal-ventral thickness of the hindpaw, as we have previously described [15]. Before baseline testing the bilateral hindpaws were tattooed with a 2 to 3 mm spot on the dorsal skin over the midpoint of the third metatarsal. For laser measurements each rat was briefly anesthetized with isoflurane and then held vertically so the hindpaw rested on a table top below the laser. The paw was gently held flat on the table with a small metal rod applied to the top of the ankle joint. Using optical triangulation, a laser with a distance measuring sensor was used to determine the distance to the table top and to the top of the hindpaw at the tattoo site and the difference was used to calculate the dorsal-ventral paw thickness. The measurement sensor device used in these experiments (4381 Precicura, Limab, Goteborg, Sweden) has a measurement range of 200 mm with a 0.01 mm resolution.

### Homogenization procedure and enzyme immunoassay for TNF-α, IL-1β, IL-6 and NGF

Rat hindpaw dorsal skin was collected after behavioral testing or at time points as indicated and frozen immediately on dry ice. Skin tissue was cut into fine pieces in ice-cold phosphate buffered saline (PBS), pH 7.4, containing protease inhibitors (aprotinin (2 μg/ml), leupeptin (5 μg/ml), pepstatin (0.7 μg/ml), and PMSF (100 μg/ml); Sigma, St. Louis, MO, USA) followed by homogenization using a rotor/stator homogenizer. Homogenates were centrifuged for 5 minutes at 14,000 g, and at 4°C. Supernatants were transferred to fresh pre-cooled Eppendorf tubes. Triton X-100 (Boehringer Mannheim, Germany) was added at a final concentration 0.01%. The samples were centrifuged again for 5 minutes at 14,000 g at 4°C. The supernatants were aliquoted and stored at −80°C. TNF-α, IL-1β, and IL-6 protein levels were determined using EIA kits (R&D Systems, Minneapolis, MN, USA). The NGF concentrations were determined using the NGF Emax® ImmunoAssay System kit (Promega, Madison, WI, USA) according to the manufacturer’s instructions. The optical density (OD) of the reaction product was read on a microplate reader at 450 nm. The concentrations of TNF-α, IL-1β, IL-6, and NGF proteins were calculated from the standard curve at each assay. Positive and negative controls were included in each assay. Each protein concentration was expressed as pg/mg total protein. Total protein contents in all tissue extracts were measured by the Coomassie Blue Protein Assay Kit (Pierce, Rockford, IL, USA).

### Tissue processing and keratinocyte immunofluorescence confocal microscopy

Animals were euthanized and immediately perfused with 4% paraformaldehyde (PFA) in PBS, pH 7.4, via the ascending aorta; the hindpaw skin including sub-dermal layers was removed and post-fixed in 4% PFA for 2 h, and then the tissues were treated with 30% sucrose in PBS at 4°C before embedding in the optimal cutting temperature compound (OCT) (Sakura Finetek USA, Inc., Torrance, California, USA). Following embedding, 10-μm thick slices were made using a cryostat, mounted onto Superfrost microscope slides (Fisher Scientific, Pittsburgh, PA, USA), and stored at −80°C.

To examine the effects of intraplantar SP on inflammatory mediator production and NK1 receptor expression in epidermal keratinocytes, double immunolabeling was performed as previously described [8]. Briefly, frozen skin sections were permeabilized and blocked with PBS containing 10% donkey serum and 0.3% Triton X-100, followed by exposure to the primary antibodies overnight at 4°C in PBS containing 2% serum. Upon detection of the first antigen, a primary antibody from a different species against the second antigen was applied to the sections and visualized using an alternative fluorophore-conjugated secondary antibody. Sections were then rinsed in PBS and incubated with fluorophore-conjugated secondary antibodies against the immunoglobulin of the species from which the primary antibody was generated. After three washes, the sections were mounted with anti-fade mounting medium (Invitrogen, Grand Island, NY, USA). With regard to primary antibodies, goat anti-rat IL-1β (R&D Systems, 1:200), goat anti-rat NGF-β (R&D Systems, 1:100), rabbit anti-rat NK1 receptor (Sigma-Aldrich, diluted 1:8000) and monoclonal mouse anti-rat keratin (clone AE1/AE3) (Thermo Fisher Scientific, Waltham, MA 02454, USA, diluted 1:50) were used. Double-labeling immunofluorescence was performed with a series of conjugated secondary antibodies (Jackson ImmunoResearch Laboratories), that is, donkey anti-mouse IgG (1:500) conjugated with fluorescein isothiocyanate (FITC) and donkey anti-goat IgG (1:500) conjugated with cyanine dye 3 (Cy3) for co-immunostaining of keratin and IL-1β or NGF, and donkey anti-mouse IgG (1:500) conjugated with Cy3 and donkey anti-rabbit IgG (1:500) conjugated with FITC for co-staining of keratin and NK1 receptor, respectively.

To assess LY303870 effects on epidermal thickness at 4 weeks post-fracture, the sections of hindpaw skin were permeabilized and blocked as described above, followed by exposure to monoclonal anti-rat keratin (clone AE1/AE3) (Thermo Fisher Scientific, diluted 1:50) overnight at 4°C in PBS containing 2% serum. The primary antibody was detected using FITC-conjugated donkey anti-mouse IgG (H + C) antibody (Jackson Immuno Reasearch Laboratories, West Grove, PA, USA, diluted 1:500). Images were obtained using confocal microscopy (Zeiss LSM/510 Upright 2 photon; Carl Zeiss, Thornwood, NY, USA) and stored on digital media. Control experiments included incubation of slices in primary and secondary antibody-free solutions both of which led to low intensity non-specific staining patterns in preliminary experiments (data not shown).

### *In vivo* bromodeoxyuridine (BrdU) labeling and BrdU immunohistochemistry

Labeling with BrdU was done to evaluate keratinocyte proliferation. At 3 weeks after tibial fracture, animals were injected intraperitoneally (i.p.) once daily with 50 mg/kg BrdU (Sigma-Aldrich) for 8 days [28]. Hindpaw skin was harvested and fixed one day after the last injection and processed for immunostaining. Skin sections were pretreated in 2 N HCl for 30 minutes at 37°C, followed by neutralization in 0.1 M borate buffer (pH 8.5) for 10 minutes and blocking with 10% normal donkey serum for 1 h at room temperature, after which immunohistochemistry was performed using a rat anti-BrdU monoclonal antibody (1:300, Accurate Chemical, WESTBURY, NY, USA) and donkey anti-rat fluorescein isothiocyanate secondary antibody (1:400, Jackson Immuno Research Laboratories). After three rinses with PBS, the sections were immunostained with the monoclonal anti-rat keratin as mentioned above. BrdU immunostaining was observed using a Leica DM 2000 fluorescent microscope and imaged using a Spot Camera (version 4.0.8, Diagnostic Instruments, Sterling Heights, MI, USA). The number of BrdU-positive cells was counted, specifically those in keratin-positive cells in the area of the epidermis with a minimum of six sections per animal from seven intact and five fractured animals. Cell densities were calculated by dividing cell numbers by the area. Representative images were obtained using confocal microscopy (Zeiss LSM/510 Upright 2 photon; Carl Zeiss).

### Microcomputed tomography (μCT)

*Ex vivo* scanning was performed for assessment of trabecular and cortical bone architecture using micro computer tomography (μCT) (VivaCT 40, Scanco Medical AG, Basserdorf, Switzerland). Specifically, trabecular bone architecture was evaluated at the distal femur and cortical bone morphology was evaluated at the midshaft femur. CT images were reconstructed in 1024 × 1024 pixel matrices for distal femur and mid-femur samples and stored in 3-dimensional arrays. The resulting grayscale images were segmented using a constrained Gaussian filter to remove noise, and a fixed threshold (25.5% of the maximal grayscale value for the vertebrae and the distal femur, and 35% for the mid-femoral cortical bone) was used to extract the structure of the mineralized tissue. The μCT parameters were set at threshold = 255, σ = 0.8, support = 1 for vertebral samples, threshold = 255, σ = 0.8, support = 1 for distal femur, and threshold = 350, σ = 1.2, and support = 2 for mid-femur evaluation analysis. A single operator outlined the trabecular bone region within the distal femur, the vertebral body and the cortical bone region in the mid-femoral shaft. The trabecular bone region was manually identified and all slices containing trabecular bone between the growth plates were included for analysis. In the distal femur 150 transverse slices of 21 μm thickness (21-μm isotropic voxel size) encompassing a length of 3.15 mm were acquired, but only 100 slices encompassing 2.1 mm of the distal femur were evaluated, starting where the growth plate bridge across the middle of the metaphysis ends. The region of interest (ROI) was manually outlined on each CT slice, extending proximally from the growth plate. The bone parameters analyzed included the bone volume fraction (BV/TV) (%), trabecular number (TbN) (mm^-1^), trabecular thickness (TbTh) (μm), trabecular separation (TbSp) (μm), and connectivity density (ConnD) (1/mm^3^). At the femoral mid-shaft, 10 transverse CT slices were obtained, each 21 μm thick totaling 0.21 mm in length (21 μm isotropic voxel size) and these were used to compute the cortical bone area (Bar) (mm^2^), total cross sectional area (TtAr) (mm^2^), medullary area (MeAr) (mm^2^), cortical thickness (CtTh) (μm), bone perimeter (BPm) (mm), and relative cortical bone area (BAr/TtAr) (%).

### Study design

To determine the time course of SP-induced pain behavior and signs of inflammation, normal rats received hindpaw intraplantar injection of SP (0, 10, 25, and 60 μg) in 50 μl of 0.9% saline. Baseline determinations were made of bilateral hindpaw mechanical nociceptive withdrawal thresholds to von Frey fibers, hindpaw temperature, and hindpaw thickness, and then the rats were injected and retested for behavior at 0.5, 1, 3, 6, 24, 48, and 72 h after SP injection (n = 6 per cohort). Based on the results of this behavioral study, a 25 μg dose of SP was selected for evaluating the time course of SP-induced inflammatory mediator expression. After normal rats underwent intraplantar injection with 25 μg SP in 50 μl of 0.9% saline, the hindpaw skin was collected at 0, 1, 3, 6, 24, and 48 h after injection for TNF-α, IL-1β, IL-6, and NGF protein level determination by EIA (n = 8 per cohort).

The effects of SP (25 μg) intraplantar injection on keratinocyte inflammatory mediator and NK1 receptor expression were evaluated by immunofluorescence confocal microscopy. Normal rats received hindpaw intraplantar injections with 25 μg SP, and then the injected hindpaw skin was collected at 0, 1, 3, and 6 h post-injection for immunostaining with anti-IL-1β, NGF, or NK1 primary antibody.

To evaluate the contribution of NGF signaling in SP-evoked allodynia, the NGF inhibitor muMab 911 (10 mg/kg) or vehicle was administered via i.p. injection in normal rats. Three days later, baseline hindpaw von Frey thresholds, temperature, and paw thickness were determined, and then the rats received an intraplantar injection with 25 μg SP. At 0.5, 1, 3, 6, 24, 48, and 72 h post-injection the animals underwent repeat tests.

To test the hypothesis that local SP signaling induces hindpaw pain and inflammation in the CRPS tibial fracture model, fracture rats were treated with either a systemic or locally administered NK1 receptor antagonist (LY303870). The rats were divided into four cohorts (n = 8 to 16 rats per cohort). Three cohorts underwent right distal tibial fracture with hindlimb cast immobilization for 4 weeks. The day after cast removal, all rats underwent bilateral hindpaw tests for von Frey thresholds, unweighting, warmth, and edema. One fracture cohort had no treatment, one cohort received daily i.p. injections of LY303870 (20 μg/kg) for 8 days prior to cast removal, and one cohort received a single intraplantar injection of LY303870 (50 μg/50 μl 0.9% saline) the day after cast removal. At one hour after intraplantar injection of LY303870 the rats were tested.

To evaluate the role SP signaling plays in post-fracture keratinocyte proliferation in the injured limb, tibial fracture rats were treated with vehicle or LY303870 (20 mg/kg i.p.) daily for 8 days prior to cast removal. All rats were injected with BrdU (50 mg/kg i.p.) daily for 8 days prior to cast removal. After cast removal the hindpaw skin was harvested bilaterally for co-immunostaining with anti-BrdU (DNA synthesis marker) and anti-keratin (keratinocyte marker) antibodies. Confocal microscopy was used to measure the number of BrdU-positive cells in the epidermis (n = 9 rats for the control, ipsilateral fracture (FX-IPSI), and contralateral fracture (FX-CONTRA) cohorts, and n = 4 for the LY303870 treatment group), and to measure epidermal thickness (n = 7 per cohort).

To test the hypothesis that SP signaling mediates fracture-induced inflammatory mediator expression in the hindpaw skin, three cohorts of rats were evaluated; controls, vehicle-treated fractured rats, and LY303870-treated fractured rats (n = 13 to 16 per cohort). LY303870 (20 mg/kg) was given by i.p. injection daily for 8 days prior to cast removal. After the casts were removed at 4 weeks post-fracture the animals were euthanized and the hindpaw skin collected for TNF-α, IL-1β, IL-6, and NGF protein level determination by EIA. The bilateral femurs were collected for *ex vivo* μCT of the proximal metaphyseal trabecular and the mid-femoral cortical bone to test the hypothesis that facilitated-SP signaling contributes to periarticular bone loss after fracture.

### Statistical analysis

Statistical analysis was accomplished using a two-way analysis of variance (ANOVA) followed by Bonferroni post hoc tests for the time course of the SP injection at various concentrations. One-way ANOVA was employed followed by Neuman-Keuls multiple comparison test to compare among the control, fractured and LY303870-treated fractured rats. All data are presented as the mean ± standard error (SE) of the mean, and differences are considered significant at a *P*-value less than 0.05 (Prism 5, GraphPad Software, San Diego, CA, USA).

Hindpaw temperature, thickness, and mechanical nociceptive thresholds data were analyzed as the difference between the treatment side and the contralateral untreated side. Right hindpaw weight bearing data were analyzed as a ratio between the right hindpaw weight and the sum of the right (R) and left (L) hindpaws values ((2R/(R + L)) × 100%).

## Results

### Time course and dosage for SP-induced allodynia, warmth, and edema in the hindpaw

**Figure 1:**
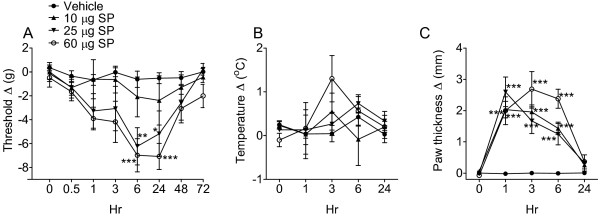
**Intraplantar injection of 10, 25, or 60 μg of substance P (SP) into the hindpaw of a normal rat dose-dependently induced mechanical allodynia to von Frey testing.** (**A**) Allodynia developed slowly, peaking at 6 h post-injection, and gradually resolving over 48 h. (**B**) There were no changes in hindpaw temperature after injecting any dose of SP. (**C**) Even the lowest dose of SP (10 μg) evoked hindpaw edema within 5 minutes, peaking at 1 h, and then gradually resolving over 24 h. Measurements for (**A**), (**B**), and (**C**) represent the difference between the injected side and the contralateral paw; thus, a positive value represents an increase in temperature or paw thickness on the injected side, and a negative value represents a decrease in mechanical nociceptive thresholds on the injected side. Data were analyzed to compare SP injection vs. vehicle injection cohorts at various time points by two-way analysis of variance (ANOVA) followed by Bonferroni post hoc test. *P < 0.05, **P < 0.01 and ***P < 0.001, SP- vs. vehicle-injected rats at indicated time points (n = 6 for each injection group). (**B**) There was no significant difference in hindpaw temperature between SP-injected and vehicle-injected rats at any time point. (**C**) Injection of SP at all concentrations (10, 25, and 60 μg) significantly evoked edema at all three time points of 1 h, 3 h and 6 h (***P < 0.001 SP- vs. vehicle-injected cohorts).

To determine the time course of SP-induced pain behavior and signs of inflammation, normal rats received an intraplantar SP (0, 10, 25, and 60 μg) injection, and then hindpaw von Frey thresholds, temperature, and thickness were determined at 0, 0.2, 1, 3, 6, 24, and 48 h post-injection (Figure 1). Higher doses of SP (25 and 60 μg) caused von Frey allodynia that developed slowly, peaking at 6 h and resolving by 48 h. SP injection had no effect on hindpaw temperature, but did induce a rapid onset of edema at all doses tested (10, 25 and 60 μg), that peaked at 1 h or 3 h and resolved by 24 h. The discrepant time courses and doses required to induce allodynia and edema suggest that different mechanisms mediate these peripheral effects of SP.

### Time course for SP-induced TNF-α, IL-1β, and IL-6, and NGF expression in hindpaw skin keratinocytes

**Figure 2:**
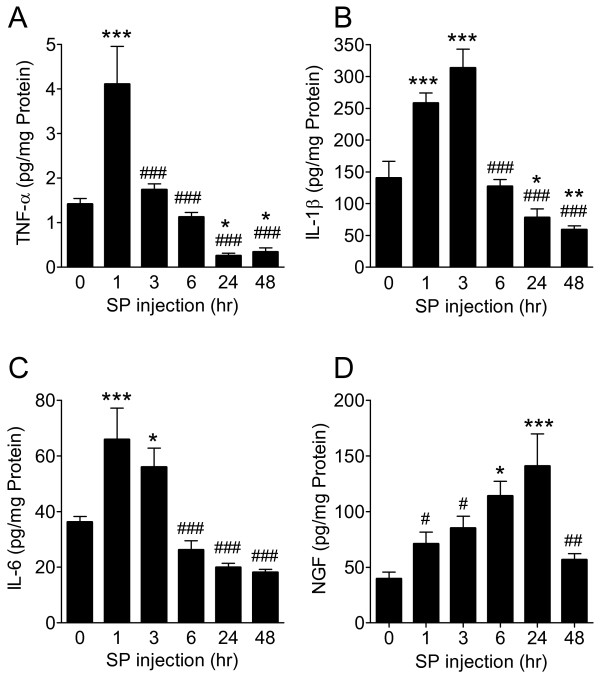
**Cytokine TNF-α (A), IL-1β (B), IL-6 (C) and nerve growth factor (NGF) (D) production in hindpaw skin was measured by EIA assay at various time points after intraplantar injection of 25 μg of substance P (SP).** Assays were performed at 0, 1, 3, 6, 24, and 48 h. SP injection induced a significant increase in all inflammatory mediators compared to baseline levels. TNF-α (**A**) was elevated at just 1 h after injection (compared to controls), IL-1β (**B**) and IL-6 (**C**) cytokine levels were elevated at 1 h and 3 h post-injection, respectively, and NGF (**D**) levels gradually increased over 24 h post-injection, dropping to baseline at 48 h. Data are expressed as mean values (pg/mg protein) ± standard error (SE) (n = 8 per cohort) and analyzed using one-way analysis of variance (ANOVA) followed by Neuman-Keuls multiple comparison test to compare different time points. *P < 0.05, **P < 0.01 and ***P < 0.001 vs. baseline at 0 h. *#P* < 0.05, ##*P* < 0.01 and ###*P* < 0.001 vs. peak values.

**Figure 3:**
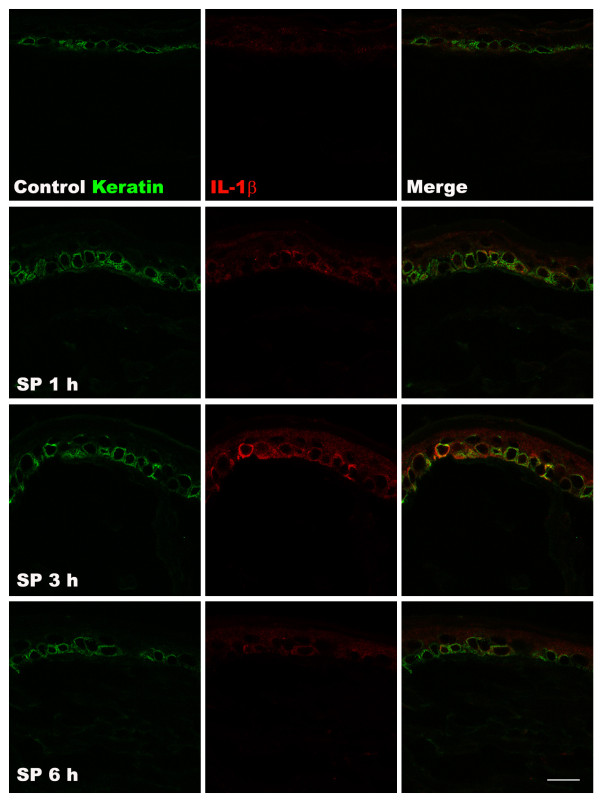
**Immunofluorescence confocal microscopy for keratin protein and IL-1β in the hindpaw skin after intraplantar injection of substance P (SP).** Kertain protein (a keratinocyte marker) (green), and IL-1β (red) are shown in control rats (top row of panels), and at 1 h (second row), 3 h (third row), and 6 h (fourth row) after intraplantar injection of 25 μg SP. Increased IL-1β staining was observed in keratinocytes at 1 h and 3 h after SP injection, resolving by 6 h, which corresponds to the time course observed using EIA assays in hindpaw skin homogenates (Figure 2). Scale bar = 20 μm.

**Figure 4:**
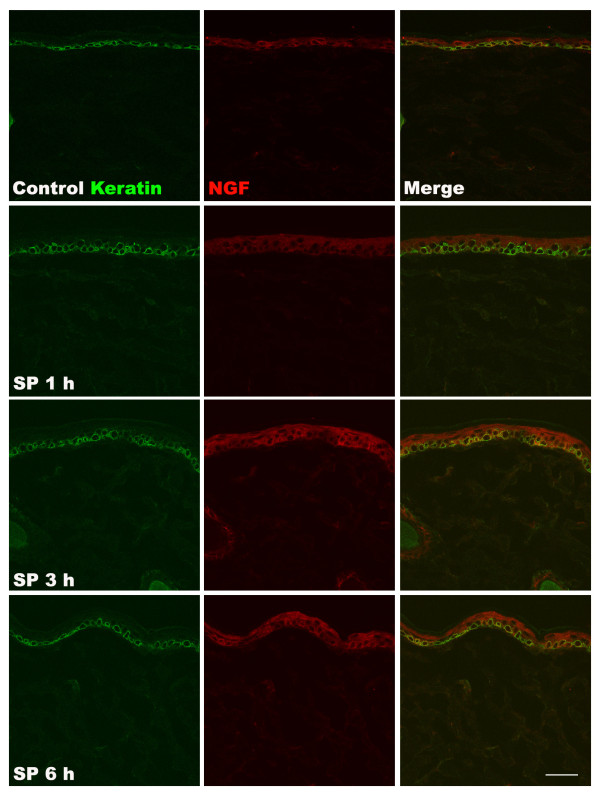
**Co-immunostaining for keratin protein and nerve growth factor (NGF) in the hindpaw skin after intraplantar injection of substance P (SP).** Keratin protein (green) and NGF (red) are shown in control rats (top row of panels), and at 1 h (second row), 3 h (third row), and 6 h (fourth row) after intraplantar injection of 25 μg SP. Increased NGF staining was observed in keratinocytes at 1, 3 and 6 h after SP injection, corresponding to the prolonged allodynia observed after SP injection (Figure 1). Scale bar = 40 μm.

**Figure 5:**
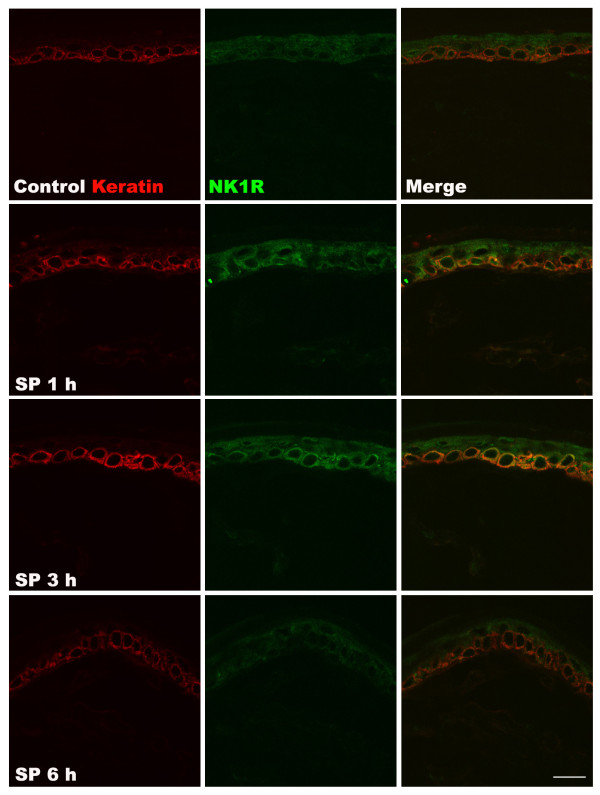
**Labeling for keratin protein and the NK1 receptor (NK1R) in the hindpaw skin after intraplantar injection of substance P (SP).** Keratin protein (red) and NK1R (green) are shown in control rats (top row of panels), and at 1 h (second row), 3 h (third row), and 6 h (fourth row) after intraplantar injection of 25 μg SP. Increased NK1R staining was observed in keratinocytes at 1 h and 3 h after SP injection, resolving by 6 h post-injection. These results suggest that facilitated SP signaling is not responsible for the prolonged hyperalgesia observed after SP injection (Figure 1). Scale bar = 20 μm.

To further elucidate the mechanisms responsible for SP-induced pain behavior, TNF-α, IL-1β, IL-6, and NGF protein levels in hindpaw skin were determined by EIA at various time points after SP (25 μg) intraplantar injection (Figure 2). TNF-α expression was upregulated at 1 h post-injection, IL-6 and IL-1β levels were increased at 1 h and 3 h post-injection, respectively, and NGF expression gradually increased from 1 h to 24 h post-injection, resolving by 48 h. Interestingly, the time course of post-injection NGF upregulation most closely paralleled the time course observed for SP-induced allodynia. When the hindpaw skin was harvested at various time points after SP (25 μg) injection for immunostaining with IL-1β antibody, increased IL-1β protein was observed in keratinocytes at 1 h and 3 h after SP injection, resolving by 6 h, which corresponds to the time course observed using EIA assays on skin homogenates (Figure 3). Similarly, when hindpaw skin was immunostained for NGF, increased levels of NGF were observed in the keratinocytes at 1, 3, and 6 h after SP injection (Figure 4). In addition, an experiment was carried out to examine NK1 receptor expression after SP injection. Immunostaining demonstrated increased NK1 receptor protein in the keratinocytes at 1 h and 3 h after SP injection, resolving by 6 h (Figure 5).

### Effects of anti-NGF antibody on SP-evoked nociceptive and vascular abnormalities

**Figure 6:**
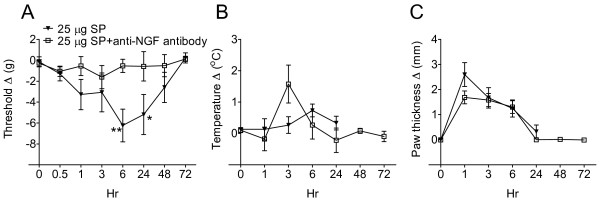
**This experiment tested the hypothesis that the delayed onset, long lasting allodynia evoked by intraplantar injection of substance P (SP) was mediated by increased nerve growth factor (NGF) expression in skin keratinocytes.** Rats (n = 8 per cohort) were treated with either anti-NGF antibody (10 mg/kg i.p. muMab 911) or vehicle, 3 days prior to undergoing intraplantar SP (25 μg) injection. (**A**) SP injection induced a slowly developing von Frey allodynia that peaked at 6 h post-injection, but SP-induced allodynia was blocked by pretreatment with anti-NGF. (**B**) There were no changes in hindpaw temperature after injecting SP. (**C**) Anti-NGF pretreatment had no effect on SP-evoked hindpaw edema. Measurements for (**A**), (**B**), and (**C**) represent the difference between the injected side and the contralateral paw, thus a positive value represents an increase in temperature or paw thickness on the injected side; a negative value represents a decrease in mechanical nociceptive thresholds on the injected side. Behavioral data after SP injection were analyzed by two-way analysis of variance (ANOVA) followed by Bonferroni post hoc test to compare differences between rats pretreated with and without anti-NGF antibody at indicated time points. *P < 0.05 and **P < 0.01 vehicle vs. anti-NGF antibody pretreatments.

The effects of anti-NGF treatment on SP-evoked hindpaw mechanical sensitivity, warmth, and edema were monitored for 72 h. Figure 6 illustrates that von Frey nociceptive thresholds in the right hindpaw were reduced after SP (25 μg) intraplantar injection but anti-NGF antibody treatment (muMab 911, 10 mg/kg i.p., given 3 days prior to testing) completely blocked the development of this mechanical allodynia. Anti-NGF antibody had no effect on hindpaw temperature or on SP-evoked hindpaw edema.

### Effects of systemic or localized LY303870 treatment on fracture-induced nociceptive and vascular abnormalities

**Figure 7:**
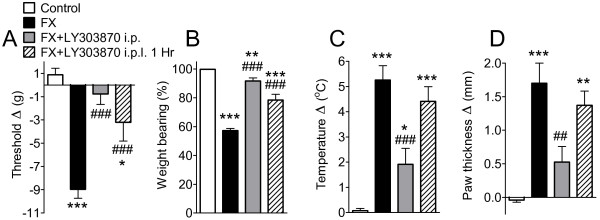
**Three cohorts of rats underwent right distal tibial fracture and hindlimb cast immobilization for four weeks.** After baseline testing, rats were divided into 4 groups**.** One fracture (FX) group had no other treatment (FX, n = 16), another fracture group underwent daily intraperitoneal (i.p.) injections with the NK1 receptor antagonist LY303870 (20 mg/kg i.p., FX + LY303870 i.p., n = 15) over an 8-day interval prior to cast removal, and another fracture group underwent a single intraplantar (i.p.l) injection of LY303870 (50 μg) on the day after cast removal and 1 h prior to testing (FX + LY303870 i.p.l. 1 h, n = 8). At four weeks post-fracture the 8 days of systemic LY303870 treatment reduced hindpaw mechanical allodynia (**A**), unweighting (**B**), warmth (**C**) and edema (**D**) that developed after fracture. In addition, all four tests were carried out 1 h after the intraplantar injection of LY303870. A single intraplantar injection of LY303870 also reduced allodynia and unweighting, but had no effect on warmth or edema. Measurements for (**A**), (**C**), and (**D**) represent the difference between the fracture side and the contralateral paw, thus a positive value represents an increase in temperature or thickness on the fracture side; a negative value represents a decrease in mechanical nociceptive thresholds on the affected side. Measurements for (**B**) represent weight-bearing on the fracture hindlimb as a ratio to 50% of bilateral hindlimb loading, thus a percentage lower than 100% represents hindpaw unweighting. Data were analyzed using one-way analysis of variance (ANOVA) followed by Neuman-Keuls multiple comparison test. *P < 0.05, **P < 0.01, ***P < 0.001 fracture vs. control (n = 13) values, and ^##^*P* < 0.01 and ^###^*P* < 0.001 for fracture with LY303870 treatments vs. fracture with vehicle treatment.

These experiments tested the hypothesis that SP signaling in the hindpaw skin is responsible for fracture-induced nociceptive and vascular changes. Rats underwent tibial fracture and cast immobilization for 4 weeks, then the cast was removed and on the following day the rats were tested for hindpaw allodynia, unweighting, warmth and edema. Fracture reduced von Frey nociceptive thresholds in the fractured hindpaw from 0.88 to −8.97 g (Figure 7). Untreated fracture rats unweighted the ipsilateral hindpaw by 43%. Fracture also increased hindpaw skin temperature by 5.2°C and thickness by 1.7 mm. When the NK1 receptor antagonist LY303870 (20 mg/kg/day) was injected i.p. for 8 consecutive days prior to cast removal there was a reduction in hindpaw allodynia, unweighting, warmth, and edema by 83, 80, 64, and 67%, respectively. Similarly, a single intraplantar injection of LY303870 (50 μg/50 μl saline) into the hindpaw skin of the fractured limb 1 h prior to behavioral testing also reduced hindpaw allodynia and unweighting, but had no effect on temperature or edema. These results suggest that SP signaling in the hindpaw skin contributes to the maintenance of pain behavior in the injured limb at 4 weeks post-fracture. We suspect that the discrepancy between the effects of systemic and intraplantar LY303870 in reversing post-fracture hindpaw warmth and edema reflect the brief duration of the intraplantar LY303870 injection effect. The very low dose (50 μg, or approximately 0.1 mg/kg) of LY303870 used in the intraplantar injection experiment had no behavioral effects when given systemically, and the analgesic effects observed after intraplantar LY303870 injection resolved by 2 h post-injection (data not shown), suggesting that the drug had dispersed systemically within 2 h post-injection and was no longer reaching effective concentrations in the hindpaw skin.

### Effects of LY303870 on fracture-induced hindpaw keratinocyte proliferation and epidermal hyperplasia

**Figure 8:**
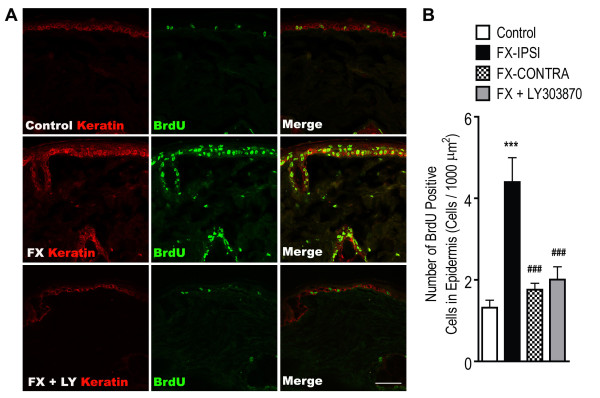
**Co-immunostaining for keratin and bromo-2-deoxyuridine (BrdU) in the hindpaw skin four weeks post-fracture.** (**A**) Top panels are images from a normal control rat, middle panels are from a fracture rat, and lower panels are from a fracture rat treated with an NK1 receptor antagonist (LY303870 20 mg/kg, i.p. daily for 8 days), showing keratin staining (red) and BrdU (a marker of DNA synthesis) staining (green). Fracture increased BrdU staining in keratinocytes, indicating increased cellular proliferation, and LY303870 treatment prevented this increase. Scale bar = 50 μm. (**B**) There was a 3.3 fold increase in BrdU-positive cell numbers at 4 weeks post-fracture in the ipsilateral fracture paw (FX-IPSI), but not in the contralateral paw (FX-CONTRA), and this increase was blocked in fractured rats treated with LY303870 (FX + LY303870). Data were analyzed using one-way analysis of variance (ANOVA) followed by Neuman-Keuls multiple comparison test. ***P < 0.001 for FX-IPSI (n = 9) vs. control (n = 9), and ^###^*P* < 0.001 for FX + LY303870 (n = 4) and FX-CONTRA (n = 9) vs. FX-IPSI cohorts.

We previously observed that distal tibial fracture led to chronic keratinocyte proliferation and epidermal hyperplasia in the hindpaw skin [8] and we hypothesized that SP-signaling was responsible for fracture-induced hindpaw keratinocyte proliferation. To test this hypothesis, BrdU was used to label DNA synthesis in proliferating keratinocytes. Figure 8 shows representative confocal images of BrdU (green) and keratin (a keratinocyte marker, red) in hindpaw skin sections from control rats, from the ipsilateral hindpaw of 4-week post-fracture rats, and from the ipsilateral hindpaw of 4 week post-fracture rats treated with LY303870 (20 mg/kg/day for 8 days just prior to cast removal). Only a few BrdU-positive cells were detected in control skin (Figure 8A, upper row of panels), but skin sections from fracture rats demonstrated a large number of BrdU-positive cells in the epidermis co-labeled for keratin, including the basal layer where new keratinocytes are generated (Figure 8A, middle row of panels). Interestingly, LY303870 treatment blocked the fracture-induced increase in BrdU-positive cell numbers in the epidermis (Figure 8A, bottom row of panels). Quantitation of the BrdU-positive cells in hindpaw skin revealed a 2.3 fold increase in BrdU-positive cells in the fractured limb (FX-IPSI) compared to the control (Figure 8B). No increase in BrdU-positive cells was observed in the hindpaw contralateral to the fractured limb (FX-CONTRA). Furthermore, LY303870 treatment reversed the fracture-evoked increase of BrdU-positive cell number in the epidermis (Figure 8B).

**Figure 9:**
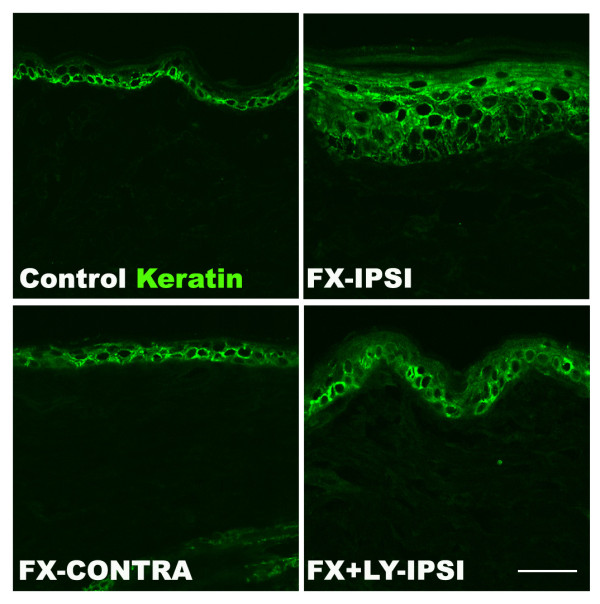
**Immunostaining for keratin in the hindpaw skin four weeks post-fracture.** Panels exhibit confocal images from the hindpaw skin of a normal control rat, the ipsilateral hindpaw skin of a fracture rat (FX-IPSI), the contralateral hindpaw skin of a fracture rat (FX-CONTRA), and the ipsilateral hindpaw skin of a fracture rat treated with an NK1 receptor antagonist (LY303870 20 mg/kg, i.p. daily for 8 days, FX + LY-IPSI), showing staining for keratin (green). Scale bar = 25 μm. There was an increase in epidermal thickness, indicating increased keratinocyte proliferation, at 4 weeks post-fracture in the fracture paw (FX-IPSI), but not in the contralateral paw (FX-CONTRA), and this increase was blocked in fractured rats treated with LY303870 (FX + LY-IPSI).

To evaluate epidermal thickening after fracture, hindpaw skin sections were stained for keratin (green) (Figure 9). Consistent with the BrdU results, we observed that fracture increased epidermal thickness and that the treatment with LY303870 for 8 days prior to cast removal blocked epidermal thickening. These results support the hypothesis that SP signaling is responsible for fracture-induced keratinocyte proliferation and epidermal hyperplasia in the hindpaw skin.

### Effects of LY303870 on fracture-induced TNF-α, IL-1β, IL-6, and NGF expression in hindpaw skin

**Figure 10:**
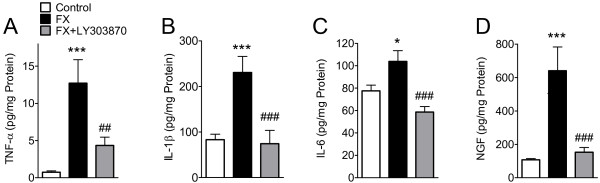
**Cytokine TNF-α (A), IL-1β (B), IL-6 (C) and nerve growth factor (NGF) (D) levels in hindpaw skin were measured by EIA assay at four weeks post-fracture.** Rats were treated with vehicle (FX, n = 16) or the NK1 receptor antagonist LY303870 (20 mg/kg i.p. daily for 8 days just prior to cast removal, FX + LY303870, n = 15). Fracture induced an increase in all inflammatory mediators compared to controls and this was blocked in the LY303870-treated fractured rats. Data are expressed as mean values (pg/mg protein) ± standard error (SE). Data were analyzed using one-way analysis of variance (ANOVA) followed by Neuman-Keuls multiple comparison test. *P < 0.05 and ***P < 0.001 vs. untreated controls (n = 13), ^##^*P* < 0.01 and ^###^*P* < 0.001 for FX + LY303870 vs. fracture.

To elucidate the involvement of SP signaling in fracture-induced upregulation of TNF-α, IL-1β, IL-6, and NGF, fractured rats were injected with LY303870 (20 mg/kg/day, i.p.) or vehicle for 8 consecutive days prior to behavioral testing and skin harvesting at 4 weeks post-fracture. Cytokines and NGF were determined by EIA of hindpaw skin homogenates. Figure 10 illustrates that TNF-α, IL-1β, IL-6 and NGF protein levels in the hindpaw skin were increased by 1593, 176, 33.9, and 495%, respectively, at 4 weeks after tibial fracture. LY303870 treatment reversed the fracture-induced upregulation of these proinflammatory cytokines and NGF, by 69.9, 105.9, 171.6, and 91.5%, respectively, indicating that SP signaling plays a critical role in post-fracture inflammatory mediator expression.

### Effects of LY303870 on fracture-induced bone loss

At 4 weeks post-fracture there was significant reduction in trabecular % BV/TV in the distal femur ipsilateral, but not contralateral to the tibial fracture (n = 10 per cohort) (Table 1). Distal femoral TbTh and ConnD were also reduced after fracture. No changes were observed in% BAr/TtAr after fracture. Treatment with the NK1 receptor antagonist LY303870 (20 mg/kg/day i.p. for 8 days just prior to cast removal) had no effect on bone loss in the distal femur, suggesting that SP signaling through the NK1 receptor does not contribute to the periarticular trabecular bone loss observed after tibial fracture.

**Table 1:** **Effects of fracture on trabecular and cortical bone parameters assessed by*ex vivo*μCT**

	**Control**	**Fracture ipsilateral**	**Fracture + LY ipsilateral**	**Fracture contralateral**	**Fracture + LY contralateral**
Distal femur					
BV/TV (%)	12.5 ± 0.6	6.60 ± 1.22***	7.31 ± 0.72***	10.63 ± 1.27	11.13 ± 0.84*
TbN (mm^-1^)	2.78 ± 0.08	2.38 ± 0.17	2.43 ± 0.14	2.91 ± 0.17	2.87 ± 0.15
TbTh (μm)	65.3 ± 1.1	57.6 ± 2.2***	62.1 ± 1.2***	63.2 ± 2.2	66.5 ± 1.2
TbSp (μm)	372 ± 11	441 ± 31	428 ± 25	358 ± 22	360 ± 18
ConnD (1/mm^3^)	40.0 ± 2.7	14.0 ± 4.3***	14.7 ± 2.5**	29.5 ± 5.2*	29.4 ± 3.8*
Femur midshaft					
BAr (mm^2^)	7.36 ± 0.13	7.42 ± 0.13	7.34 ± 0.21	7.27 ± 0.14	7.34 ± 0.20
TtAr (mm^2^)	13.4 ± 0.4	13.4 ± 0.3	13.1 ± 0.3	13.2 ± 0.3	13.1 ± 0.3
MeAr (mm^2^)	6.11 ± 0.29	5.99 ± 0.22	5.74 ± 0.24	5.96 ± 0.21	5.72 ± 0.23
CtTh (μm)	703 ± 6	720 ± 7	723 ± 18	707 ± 8	725 ± 20
BPm (mm)	23.9 ± 0.5	22.4 ± 0.4*	21.3 ± 0.4**	22.6 ± 0.4	21.9 ± 0.3**
BAr/TtAr (%)	54.8 ± 0.9	55.4 ± 0.7	56.2 ± 1.3	55.0 ± 0.5	56.2 ± 1.3

Statistical significance determined from one-way analysis of variance (ANOVA) followed by post hoc Newman-Keuls test. All data are expressed as means ± standard error of the mean. *P < 0.05, **P < 0.01 and ***P < 0.001 vs. controls.

BV: bone volume; TV: total volume; TbN: trabecular number; TbTh: trabecular thickness; TbSp: trabecular separation; ConnD: connectivity density; Bar: bone area: TtAr: total cross sectional area; MeAr: medullary area; CtTh: cortical thickness; BPm: bone perimeter; LY: LY303870.

## Discussion

Substantial evidence exists to suggest that neuropeptide mediated mechanisms support pain and edema in CRPS. Using a rat model involving tibial fracture and cast immobilization that faithfully reproduces many of the features of CRPS type I in humans, we previously showed that SP signaling through the NK1 receptor was required for the full manifestation of the syndrome [2]. Similar findings were reported using a sciatic section model of CRPS type II, a condition defined as involving frank damage to a peripheral nerve [27]. Unknown, however, is the mechanism through which SP acts to cause the CRPS-related changes. Additional data suggest that cytokines such as TNF-α, IL-1β, and IL-6, as well as the neurotrophin NGF, might contribute to pain and vascular changes in CRPS [4,5,7,8]. Therefore, we set out to determine if SP-supported production of any of these mediators contributes to the CRPS phenotype in the rat fracture/cast model. We found that 1) the time course of NGF production after intradermal CRPS injection most closely matched the time course of SP-mediated nociceptive sensitization; 2) SP-stimulated NGF production was localized to the epidermal keratinocytes; 3) anti-NGF could completely block the nociceptive changes but not the vascular effects of SP; 4) the blockade of SP signaling using the NK1 receptor antagonist LY303870 effectively reduced nociceptive and vascular manifestations of CRPS in the fracture/cast model, but not bone-related changes; 5) the keratinocyte proliferation characteristic of CRPS in the rat model was dependent on SP signaling, and 6) LY303870 blocked the production of NGF as well as several cytokines in the skin of the fracture/cast rats. Together these results indicate that while SP has many effects related to the manifestations of CRPS, it is the stimulation of NGF production that seems to be most closely related to supporting nociceptive sensitization.

Neuropeptides, most notably SP, have been implicated in the pathophysiology of CRPS in both humans and, correspondingly, in animal models. Analysis of venous blood showed elevation of SP in CRPS patients [29]. When SP was administered to CRPS skin through microdialysis fibers, enhanced levels of protein extravasation were seen relative to the responses in unaffected individuals [11]. Furthermore, electrical stimulation of the limbs of patients with CRPS leads to greater neuropeptide-dependent protein extravasation than in control individuals [9]. It is notable that the use of angiotensin converting enzyme inhibitors leads to an apparent increased risk for the development of CRPS [30]. These agents slow the breakdown of SP.

Previously reported animal studies support these observations. For example, animals deficient in the endogenous SP-metabolizing enzyme, neutral endopeptidase (NEP), develop greater nociceptive and limb neurogenic changes after chronic constriction injury (CCI) [31]. As mentioned above, previous experiments from our own laboratory demonstrated that NK1 antagonism inhibited nociceptive sensitization in models of both CRPS I and II [2,27]. It may also be relevant that SP-deficient mice have less robust allodynia and cytokine generation in skin after hindpaw incision [32], and that NK1 receptor antagonists have been demonstrated to be effective analgesics for post-operative pain in a clinical trial [33] (though not in trials involving other types of pain). While no clinical trial data are currently available examining NK1 receptor antagonist efficacy in CRPS, future trials might have their greatest opportunity for success in patients with acute or warm-phase CRPS syndrome where neurogenic inflammation is most prominent. This is the time period most similar to the rodent model used in these studies.

Our studies went beyond the simple description of NK1 control of specific cytokines and NGF in paw skin after SP administration and in the fracture model of CRPS. We attempted to determine which of the several mediators was most closely linked to SP’s pro-nociceptive effects. Based on the time course of production and the time course for nociceptive sensitization, NGF appeared to be the most plausible candidate. In fact, anti-NGF was able to block the pro-nociceptive effects of SP, but not the vascular effects, which follow a distinctly shorter time course. Earlier studies demonstrated that the same anti-NGF reagent blocked nociceptive sensitization but not the production of other inflammatory mediators in the fracture model [5]. The NK1 receptor antagonist also blocked NGF production in the fracture model. Furthermore, the current study demonstrated that epidermal keratinocytes are the cellular source for cutaneous NGF after SP injection. Both rodents and humans express NK1 receptors on keratinocytes [14,34]. It seems reasonable to hypothesize that NGF formed in the epidermis stimulates TrkA, expressing primary afferent neurons leading to nociceptive sensitization [35]. NGF receptors on vascular tissue are far less well described. Though data from human trials conducted on CRPS patients are not available, anti-NGF has the ability to reduce other forms of refractory chronic pain in humans including back pain and pain due to osteoarthritis [36-39]. Our data suggest that anti-NGF might be an effective therapy for the pain, but not the vascular changes associated with CRPS.

Skin is the body’s largest organ, and keratinocytes are the most common cell type in the epidermis, the skin’s outermost cellular layer. Far from performing only the function of providing a barrier against external threats, skin has a number of endocrinologic and immunologic roles. Indeed the skin has various roles in supporting pain. Recent studies of gene expression and keratinocyte function suggest that functional changes in these cells support pain in several clinical pain syndromes. For example, studies of skin biopsies from patients with both post-herpetic neuralgia and CRPS I have identified alterations in several sodium ion channel types including Na(v) 1.2, 1.2, 1.5, 1.6, 1.7 and 1.8, channels normally associated with controlling the electrical excitability of nociceptive neurons [40]. While the function of those channels in keratinocytes is somewhat unclear, recent observations describe a more plausible mechanism supporting nociception involving the expression of CGRP. The skin of patients with painful neuropathies was observed to produce the β form of CGRP aberrantly [41]. Keratinocyte release of this neuropeptide would allow it to interact with nociceptive nerve terminals in the epidermis and to act in a paracrine and autocrine fashion on other keratinocytes, providing potential mechanisms for skin to enhance nociceptive sensitivity. Likewise we have identified cytokines such as IL-1β, IL-6, TNF-α as well as NGF in the keratinocytes of mice and rats after tibial fracture and immobilization [8]. Significantly, peripherally restricted biologic antagonists to these mediators are very effective in reducing the nociceptive and related changes characteristic of CRPS I in rats [4,5,7]. In the present set of studies we were able to refine our understanding of the neuroinflammatory link between SP and NGF production at the cellular level in a fracture model of CRPS. Not only were NK1 receptors upregulated on keratinocytes after SP injection, but NGF production was strongly enhanced as well. Finally, we observed that SP affected more than just mediator production in keratinocytes, in that cellular proliferation was stimulated as well. Trophic changes involving the skin are characteristic of many patients with CRPS. Thus keratinocytes are affected in multiple ways by the release of SP.

A range of SP effects have been observed in models of acute and chronic pain. Signaling mediated by this molecule has been studied in translational fashion in some detail. While there have been disappointments in drug development efforts where blockade of SP signaling was hoped to have immediate analgesic benefits in otherwise normal patients [42], SP is still likely to support more complex biological pathways. It has been shown to be highly active in human skin, especially in the setting of CRPS. Our results suggest that we should focus more on SP’s ability to support the production of additional downstream mediators, like NGF, in indirectly supporting nociceptive sensitization. We also need to realize that changes in the skin of patients with CRPS are complex, and that there may be no single signaling pathway adequately explaining all relevant changes. Thus the principal goal to be set for future investigations in this area might be to integrate the functions of the already identified signaling molecules, systems and cascades into a coherent representation of the pathophysiology of CRPS.

## Abbreviations

ANOVA, analysis of variance; BAr, cortical bone area; BAr/TtAr, relative cortical bone area; BPm, bone perimeter; BrdU, 5-bromo-2-deoxyuridine; BV/TV, bone volume fraction; CCI, chronic constriction injury; CGRP, calcitonin gene-related peptide; ConnD, connectivity density; CONTRA, contralateral; CRPS, complex regional pain syndrome; CtTh, cortical thickness; Cy3, cyanine dye 3; FITC, fluorescein isothiocyanate; FX, Fracture; IL, interleukin; IPSI, Ipsilateral; MeAr, medullary area; NGF, nerve growth factor-β; NK1, neurokinin-1; OD, optical density; PBS, phosphate buffered saline; PFA, paraformaldehyde; ROI, region of interest; SP, substance P; TbN, trabecular number; TbSp, trabecular separation; TbTh, trabecular thickness; TNF, tumor necrosis factor; TtAr, total cross sectional area; μCT, micro computer tomography.

## Competing interests

The authors declare that they have no competing interests.

## Authors’ contributions

TW performed the biochemical assays, analyzed the data, generated figures, and contributed to the writing of the manuscript. TG generated the fracture rats, performed the behavioral experiments, analyzed the data, and generated figures. WL performed the immunohistochemistry, analyzed the data, and generated figures. SH performed μCT scanning, analyzed data and generated the table. WK participated in the design, data analysis, and editing of this manuscript. DC participated in the design, data analysis, and editing of this manuscript. All the authors have read and approved the final manuscript.

## References

[B1] Sarangi PP Ward AJ Smith EJ Staddon GE Atkins RM Algodystrophy and osteoporosis after tibial fractures J Bone Joint Surg Br 1993 75 450 452 8496220 10.1302/0301-620X.75B3.8496220

[B2] Guo TZ Offley SC Boyd EA Jacobs CR Kingery WS Substance P signaling contributes to the vascular and nociceptive abnormalities observed in a tibial fracture rat model of complex regional pain syndrome type I Pain 2004 108 95 107 10.1016/j.pain.2003.12.010 15109512

[B3] Groeneweg JG Huygen FJ Heijmans-Antonissen C Niehof S Zijlstra FJ Increased endothelin-1 and diminished nitric oxide levels in blister fluids of patients with intermediate cold type complex regional pain syndrome type 1 BMC Musculoskelet Disord 2006 7 91 10.1186/1471-2474-7-91 17137491 PMC1693561

[B4] Sabsovich I Guo TZ Wei T Zhao R Li X Clark DJ Geis C Sommer C Kingery WS TNF signaling contributes to the development of nociceptive sensitization in a tibia fracture model of complex regional pain syndrome type I Pain 2008 137 507 519 10.1016/j.pain.2007.10.013 18035493 PMC2529181

[B5] Sabsovich I Wei T Guo TZ Zhao R Shi X Li X Yeomans DC Klyukinov M Kingery WS Clark JD Effect of anti-NGF antibodies in a rat tibia fracture model of complex regional pain syndrome type I Pain 2008 138 47 60 10.1016/j.pain.2007.11.004 18083307 PMC2538487

[B6] Wei T Sabsovich I Guo TZ Shi X Zhao R Li W Geis C Sommer C Kingery WS Clark DJ Pentoxifylline attenuates nociceptive sensitization and cytokine expression in a tibia fracture rat model of complex regional pain syndrome Eur J Pain 2009 13 253 262 10.1016/j.ejpain.2008.04.014 18554967 PMC2673487

[B7] Li WW Sabsovich I Guo TZ Zhao R Kingery WS Clark JD The role of enhanced cutaneous IL-1beta signaling in a rat tibia fracture model of complex regional pain syndrome Pain 2009 144 303 313 10.1016/j.pain.2009.04.033 19473768 PMC2743308

[B8] Li WW Guo TZ Li XQ Kingery WS Clark JD Fracture induces keratinocyte activation, proliferation, and expression of pro-nociceptive inflammatory mediators Pain 2010 151 843 852 10.1016/j.pain.2010.09.026 20934254 PMC2972360

[B9] Weber M Birklein F Neundorfer B Schmelz M Facilitated neurogenic inflammation in complex regional pain syndrome Pain 2001 91 251 257 10.1016/S0304-3959(00)00445-0 11275381

[B10] Oyen WJ Arntz IE Claessens RM Van der Meer JW Corstens FH Goris RJ Reflex sympathetic dystrophy of the hand: an excessive inflammatory response? Pain 1993 55 151 157 10.1016/0304-3959(93)90144-E 8309706

[B11] Leis S Weber M Isselmann A Schmelz M Birklein F Substance-P-induced protein extravasation is bilaterally increased in complex regional pain syndrome Exp Neurol 2003 183 197 204 10.1016/S0014-4886(03)00163-8 12957502

[B12] Leis S Weber M Schmelz M Birklein F Facilitated neurogenic inflammation in unaffected limbs of patients with complex regional pain syndrome Neurosci Lett 2004 359 163 166 10.1016/j.neulet.2004.02.025 15050689

[B13] Weidner C Klede M Rukwied R Lischetzki G Neisius U Skov PS Petersen LJ Schmelz M Acute effects of substance P and calcitonin gene-related peptide in human skin–a microdialysis study J Invest Dermatol 2000 115 1015 1020 10.1046/j.1523-1747.2000.00142.x 11121135

[B14] Wei T Li WW Guo TZ Zhao R Wang L Clark DJ Oaklander AL Schmelz M Kingery WS Post-junctional facilitation of Substance P signaling in a tibia fracture rat model of complex regional pain syndrome type I Pain 2009 144 278 286 10.1016/j.pain.2009.04.020 19464118 PMC2706925

[B15] Guo TZ Wei T Kingery WS Glucocorticoid inhibition of vascular abnormalities in a tibia fracture rat model of complex regional pain syndrome type I Pain 2006 121 158 167 10.1016/j.pain.2005.12.022 16472917

[B16] Paus R Heinzelmann T Robicsek S Czarnetzki BM Maurer M Substance P stimulates murine epidermal keratinocyte proliferation and dermal mast cell degranulation in situ Arch Dermatol Res 1995 287 500 502 10.1007/BF00373436 7542862

[B17] Tanaka T Danno K Ikai K Imamura S Effects of substance P and substance K on the growth of cultured keratinocytes J Invest Dermatol 1988 90 399 401 10.1111/1523-1747.ep12456487 2450147

[B18] Saade NE Massaad CA Ochoa-Chaar CI Jabbur SJ Safieh-Garabedian B Atweh SF Upregulation of proinflammatory cytokines and nerve growth factor by intraplantar injection of capsaicin in rats J Physiol 2002 545 241 253 10.1113/jphysiol.2002.028233 12433964 PMC2290671

[B19] Zimmermann M Ethical guidelines for investigations of experimental pain in conscious animals Pain 1983 16 109 110 10.1016/0304-3959(83)90201-4 6877845

[B20] Hongo JS Laramee GR Urfer R Shelton DL Restivo T Sadick M Galloway A Chu H Winslow JW Antibody binding regions on human nerve growth factor identified by homolog- and alanine-scanning mutagenesis Hybridoma 2000 19 215 227 10.1089/02724570050109611 10952410

[B21] Halvorson KG Kubota K Sevcik MA Lindsay TH Sotillo JE Ghilardi JR Rosol TJ Boustany L Shelton DL Mantyh PW A blocking antibody to nerve growth factor attenuates skeletal pain induced by prostate tumor cells growing in bone Cancer Res 2005 65 9426 9435 10.1158/0008-5472.CAN-05-0826 16230406

[B22] Sevcik MA Ghilardi JR Peters CM Lindsay TH Halvorson KG Jonas BM Kubota K Kuskowski MA Boustany L Shelton DL Mantyh PW Anti-NGF therapy profoundly reduces bone cancer pain and the accompanying increase in markers of peripheral and central sensitization Pain 2005 115 128 141 10.1016/j.pain.2005.02.022 15836976

[B23] Shelton DL Zeller J Ho WH Pons J Rosenthal A Nerve growth factor mediates hyperalgesia and cachexia in auto-immune arthritis Pain 2005 116 8 16 10.1016/j.pain.2005.03.039 15927377

[B24] Gitter BD Bruns RF Howbert JJ Waters DC Threlkeld PG Cox LM Nixon JA Lobb KL Mason NR Stengel PW Pharmacological characterization of LY303870: a novel, potent and selective nonpeptide substance P (neurokinin 1) receptor antagonist J Pharmacol Exp Therapeut 1995 275 737 744 7473161

[B25] Hipskind PA Howbert JJ Bruns RF Cho SSY Crowell TA Foreman MM Gehlert DR Iyengar S Johnson KW Krushinski JH Li DL Lobb KL Mason NR Muehl BS Nixon JA Phebus LA Regoli D Simmons RM Threlkeld PG Waters DC Gitter BD 3-Aryl-1,2,-diacetamidopropane derivatives as novel and potent NK-1 receptor antagonists J Med Chem 1996 39 736 748 10.1021/jm950616c 8576917

[B26] Iyengar S Hipskind PA Gehlert DR Schober D Lobb KL Nixon JA Helton DR Kallman MJ Boucher S Couture R Li DL Simmons RM LY303870, a centrallyactive neurokinin-1 antagonist with a long duration of action J Pharmacol Exp Ther 1997 280 774 785 9023291

[B27] Kingery WS Davies MF Clark JD A substance P receptor (NK1) antagonist can reverse vascular and nociceptive abnormalities in a rat model of complex regional pain syndrome type II Pain 2003 104 75 84 10.1016/S0304-3959(02)00467-0 12855316

[B28] Wojtowicz JM Kee N BrdU assay for neurogenesis in rodents Nat Protoc 2006 1 1399 1405 10.1038/nprot.2006.224 17406427

[B29] Schinkel C Gaertner A Zaspel J Zedler S Faist E Schuermann M Inflammatory mediators are altered in the acute phase of posttraumatic complex regional pain syndrome Clin J Pain 2006 22 235 239 10.1097/01.ajp.0000169669.70523.f0 16514322

[B30] de Mos M Huygen FJ Stricker BH Dieleman JP Sturkenboom MC The association between ACE inhibitors and the complex regional pain syndrome: Suggestions for a neuro-inflammatory pathogenesis of CRPS Pain 2009 142 218 224 10.1016/j.pain.2008.12.032 19195784

[B31] Kramer HH He L Lu B Birklein F Sommer C Increased pain and neurogenic inflammation in mice deficient of neutral endopeptidase Neurobiol Dis 2009 35 177 183 10.1016/j.nbd.2008.11.002 19084065

[B32] Sahbaie P Shi X Guo TZ Qiao Y Yeomans DC Kingery WS Clark JD Role of substance P signaling in enhanced nociceptive sensitization and local cytokine production after incision Pain 2009 145 341 349 10.1016/j.pain.2009.06.037 19660865 PMC2746201

[B33] Dionne RA Max MB Gordon SM Parada S Sang C Gracely RH Sethna NF MacLean DB The substance P receptor antagonist CP-99,994 reduces acute postoperative pain Clin Pharmacol Ther 1998 64 562 568 10.1016/S0009-9236(98)90140-0 9834049

[B34] Kingery WS Role of neuropeptide, cytokine, and growth factor signaling in complex regional pain syndrome Pain Med 2010 11 1239 1250 10.1111/j.1526-4637.2010.00913.x 20704672

[B35] Malik-Hall M Dina OA Levine JD Primary afferent nociceptor mechanisms mediating NGF-induced mechanical hyperalgesia Eur J Neurosci 2005 21 3387 3394 10.1111/j.1460-9568.2005.04173.x 16026476

[B36] Cattaneo A Tanezumab, a recombinant humanized mAb against nerve growth factor for the treatment of acute and chronic pain Curr Opin Mol Ther 2010 12 94 106 20140821

[B37] Lane NE Schnitzer TJ Birbara CA Mokhtarani M Shelton DL Smith MD Brown MT Tanezumab for the treatment of pain from osteoarthritis of the knee N Engl J Med 2010 363 1521 1531 10.1056/NEJMoa0901510 20942668 PMC6896791

[B38] Schnitzer TJ Lane NE Birbara C Smith MD Simpson SL Brown MT Long-term open-label study of tanezumab for moderate to severe osteoarthritic knee pain Osteoarthr Cartil 2011 19 639 646 10.1016/j.joca.2011.01.009 21251985

[B39] Katz N Borenstein DG Birbara C Bramson C Nemeth MA Smith MD Brown MT Efficacy and safety of tanezumab in the treatment of chronic low back pain Pain 2011 152 2248 2258 10.1016/j.pain.2011.05.003 21696889

[B40] Zhao P Barr TP Hou Q Dib-Hajj SD Black JA Albrecht PJ Petersen K Eisenberg E Wymer JP Rice FL Waxman SG Voltage-gated sodium channel expression in rat and human epidermal keratinocytes: evidence for a role in pain Pain 2008 139 90 105 10.1016/j.pain.2008.03.016 18442883

[B41] Hou Q Barr T Gee L Vickers J Wymer J Borsani E Rodella L Getsios S Burdo T Eisenberg E Guha U Lavker R Kessler J Chittur S Fiorino D Rice F Albrecht P Keratinocyte expression of calcitonin gene-related peptide beta: implications for neuropathic and inflammatory pain mechanisms Pain 2011 152 2036 2051 10.1016/j.pain.2011.04.033 21641113 PMC3157543

[B42] Urban LA Fox AJ NK1 receptor antagonists–are they really without effect in the pain clinic? Trends Pharmacol Sci 2000 21 462 464 author reply 465 10.1016/S0165-6147(00)01578-9 11229417

